# P-2270. Spectrum of Invasive Mold Sinusitis in Adults with Hematological Malignancies

**DOI:** 10.1093/ofid/ofae631.2423

**Published:** 2025-01-29

**Authors:** Isabel C Ramirez

**Affiliations:** Hospital Pablo Tobon Uribe, Universidad de Antioquia, Medellin, Antioquia, Colombia

## Abstract

**Background:**

Invasive mold sinusitis (IMS) occurs exclusively in immunocompromised patients. In patients with hematologic malignancies, it is associated with mortality and may affect cancer-related survival by causing delay of the chemotherapy regimen.

Clinical characteristics of patients with IMS

**Figure:**
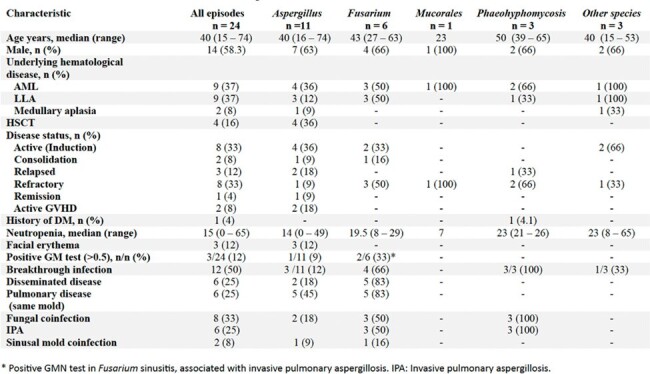

**Methods:**

The records of adult patients with hematologic malignancies and proven or probable IMS over a 10-year period at a tertiary care hospital in Medellin, Colombia were retrospectively reviewed. Clinical aspects and results were evaluated

Microbiological data of IMS

**Figure:**
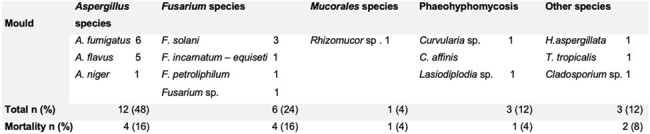

**Results:**

We identified 24 episodes of IMS in 23 patients. In all cases manifested with nasal congestion, 27% of the episodes occurred in acute leukemias, 50% in refractory disease and 22.2% in medullary aplasia and hematopoietic stem cell transplantation; 16.6% manifested as a breakthrough infection. 21 episodes were related to neutropenia at the time of diagnosis. *Aspergillus* spp. was isolated in 12 (52.1%) episodes, *Fusarium* spp. in 6 (26%). Other etiologies were *Curvularia* spp., *Hormographiella aspergillata*, *Tropicoporus tropicalis*, *Rhizomucor* spp., *Cladosporium* and *Lasiodiplodia* spp. In 5 episodes there was fungal coinfection mainly with *Aspergillus* spp. In 26% of the cases the sinusitis was related to invasive pulmonary aspergillosis (IPA). The galactomannan antigen test was positive in patients with IPA and negative in cases of isolated sinusitis. All patients underwent surgical debridement and had positive histology and microbiological isolation with morphological and/or molecular identification. 13 episodes were treated with voriconazole and the response was defined as complete and partial in 10 and 8 of the episodes respectively. 7 patients died, 5 due to refractory hematological disease and one attributable to infection as it was rhinosinusal mucormycosis.

**Conclusion:**

IMS is an expected complication of cytotoxic chemotherapy and is usually associated with profound and prolonged neutropenia. It is related to high mortality given the underlying malignant disease. Although *Aspergillus* spp. is the most common etiology, there are other molds that must be taken into account for better therapeutic guidance and therefore the need for invasive diagnosis is highlighted.

**Disclosures:**

All Authors: No reported disclosures

